# Evolutionary distance estimation and fidelity of pair wise sequence alignment

**DOI:** 10.1186/1471-2105-6-102

**Published:** 2005-04-19

**Authors:** Michael S Rosenberg

**Affiliations:** 1 Center for Evolutionary Functional Genomics, The Biodesign Institute, and the School of Life Sciences, Arizona State University, Tempe, AZ 84287-4501, USA

## Abstract

**Background:**

Evolutionary distances are a critical measure in comparative genomics and molecular evolutionary biology. A simulation study was used to examine the effect of alignment accuracy of DNA sequences on evolutionary distance estimation.

**Results:**

Under the studied conditions, distance estimation was relatively unaffected by alignment error (50% or more of the sites incorrectly aligned) as long as 50% or more of the sites were identical among the sequences (observed *P*-distance < 0.5). Beyond this threshold, the alignment procedure artificially inflates the apparent sequence identity, skewing distance estimates, and creating alignments that are essentially indistinguishable from random data. This general result was independent of substitution model, sequence length, and insertion and deletion size and rate.

**Conclusion:**

Examination of the estimated sequence identity may yield some guidance as to the accuracy of the alignment. Inaccurate alignments are expected to have large effects on analyses dependent on site specificity, but analyses that depend on evolutionary distance may be somewhat robust to alignment error as long as fewer than half of the sites have diverged.

## Background

Evolutionary distance, the number of substitutions per site separating a pair of homologous sequences since they diverged from their common ancestral sequence, is an extremely important measure in molecular evolution and comparative genomics. It is used for a wide variety of purposes, ranging from phylogenetic analysis [1,2], to estimating times of divergence [3,4], the tempo and mode of evolutionary change [5], and functional constraints [6,7]. Evolutionary distance estimation is often one of the first steps in high-throughput sequence analysis; errors in these estimates may have wide-ranging consequences on downstream analyses and conclusions.

There are many ways to estimate evolutionary distance; accuracy of various methods tends to be dependent on proper specification of the substitution model and sequence length [8,9]. One factor that has not been well examined with respect to evolutionary distance estimation, however, is alignment (although see [10-12]). Sequence alignment is an extremely common analytical tool used in comparative genomics. The purpose of alignment is to identify positions in homologous sequences that are descended from a common ancestor. Because alignment is the first step in many complex, high-throughput studies [13], it is often forgotten that alignment algorithms produce a *hypothesis *of homology (just as a phylogenetic tree is a hypothesis of evolutionary history). As with other hypotheses, these alignments may contain more or less error depending on the nature of the data. While it is widely recognized that highly divergent sequences are more difficult to align and will contain more error than less divergent sequences (e.g., [14,15]), the nature of this error appears to be underappreciated and is usually ignored.

Little attention has been paid to how errors in sequence alignment affect downstream analysis. Individual studies have shown that error in alignment can have broad effects on computational approaches to discovering functional elements [16,17] and phylogenetic analysis (e.g., [18-24]); these studies have been based on specific data sets and generally show that different results are obtained by different alignments, rather than estimating the amount of error generated by incorrect alignment.

I performed a simulation study to examine the relationship between global alignment accuracy and evolutionary distance estimation of noncoding DNA sequences. It consists of a profile of the magnitude of error one expects to find in paired sequence alignment under the simulation conditions and the comparison of evolutionary distance estimates from correct and hypothesized alignments as the true divergence increases.

## Results and discussion

**Figure 1:**
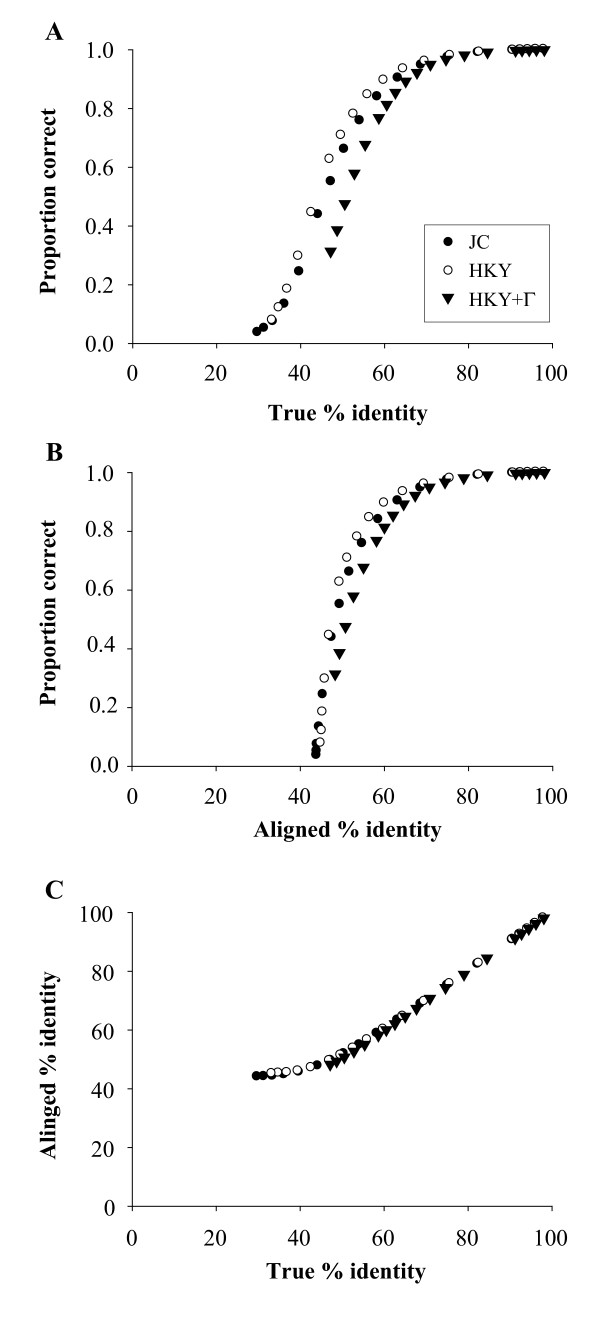
**Relationship of alignment accuracy and sequence identity**. (**A**) Proportion of sites correctly aligned versus true percent identity among the sequences. (**B**) Proportion of sites correctly aligned versus observed percent identity after alignment. (**C**) Observed versus true percent identity. JC = Jukes-Cantor model [37]; HKY = Hasegawa-Kishino-Yano model [38]; HKY + Γ = Hasegawa-Kishino-Yano plus gamma-distributed rates model. All points represent the average of 1000 simulation replicates.

Under the baseline simulation conditions, alignment accuracy (measured as the proportion of aligned sites that are truly homologous) is largely dependent on the proportion of homologous sites containing identical nucleotides. When sequence identity exceeded 80%, essentially all aligned sites (>99%) were truly homologous (Figure 1). As identity declined, the proportion of correctly aligned sites rapidly decreased. When identity reached 65%, about 90% of the aligned sites were still correct, but when identity reached 50% accuracy dropped to 30–65% (depending on the complexity of the substitution model). When fewer than 50% of truly homologous sites were identical, alignment accuracy becomes essentially zero.

A distinction needs to be made between the true identity of the sequences (the proportion of truly homologous sites in a pair of sequences containing identical nucleotides) and the aligned identity (the proportion of hypothesized homologous sites from an alignment that contain identical nucleotides). The nature of alignment algorithms is to predict homology by inserting gaps so that sites with identical nucleotides align. When true variation among sequences is large, algorithms can be quite efficient at incorrectly inferring identity. The theoretical minimum identity for a pair of sequences under the present simulation conditions is 25–26% (depending on the specific substitution model), yet Clustal yields sequences with a minimum identity of 44% (Figure 1), even for random data (similar results have been reported by others, e.g., [10,25]). The inflation in observed identity is predominantly found in sequences that truly differ by more than 50% of their sites; sequences with true identity of 50% or more have less than a 1% absolute increase in observed identity after alignment.

The results represented in Figure 1 describe the accuracy of pair wise alignment by Clustal under the specific simulation conditions and alignment parameters. While these exact profiles cannot be taken as representative of alignment accuracy for all sequences and algorithms, the shape of the curve probably does reflect a general pattern. Different evolutionary conditions and algorithms may lead to varying inflection points, but the general shape of the curve is likely to be constant (e.g., similar accuracy curves were found in [26]).

**Figure 2:**
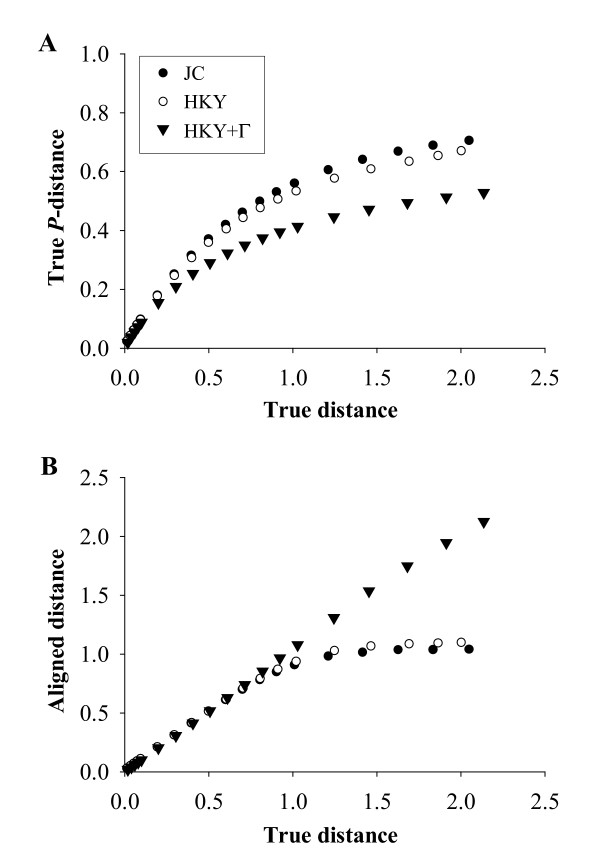
**Relationships among true and estimated evolutionary distances**. True distances were measured using the appropriate substitution model. (**A**) True *P*-distance versus true evolutionary distance. (**B**) Estimated evolutionary distance from aligned data versus true evolutionary distance.

**Figure 3:**
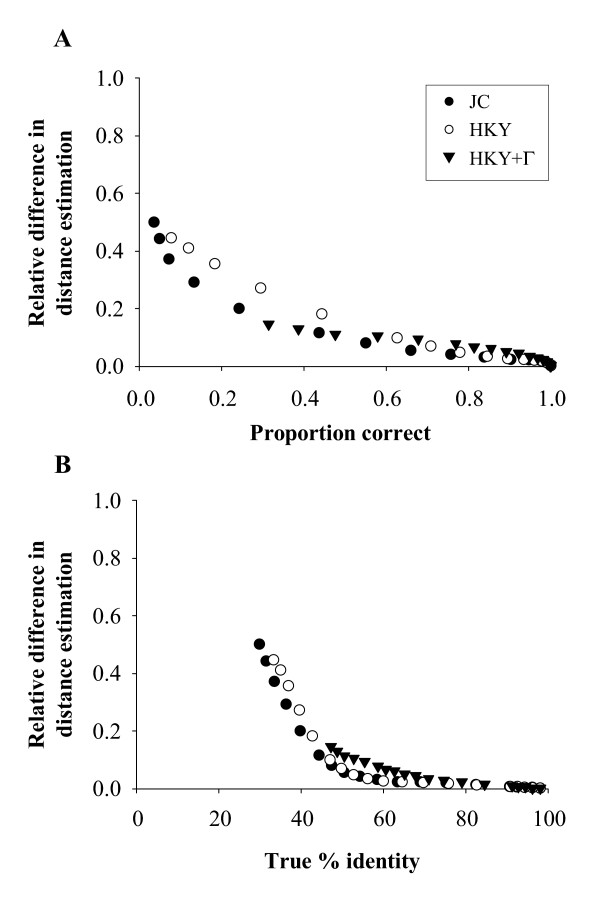
**Effect of alignment error on evolutionary distance estimation**. Relative error in evolutionary distance is measured as the absolute difference between the distance estimated from the true alignment and the distance estimated from the observed alignment, divided by the distance estimate from the true alignment. (**A**) Relative error in evolutionary distance versus proportion of correctly aligned sites. (**B**) Relative error in evolutionary distance versus true percent identity.

Up to a point, evolution distance estimation is somewhat robust to alignment error (Figures 2,3). The relative difference between evolutionary distances estimated from the true and hypothesized alignments (= |*d*_*true *_- *d*_*align*_|/*d*_*true*_) is less than 10%, even when up to 50% of the sites are aligned incorrectly (Figure 3). Distance estimates from the hypothesized alignments begin to differ to a greater extent from the true alignment only when more than half of the sites are aligned incorrectly. For the JC and HKY substitution models, alignment inaccuracy did not have an effect on distance estimation for true distances less than 1.0 (Figure 2). For HKY + Γ, alignment inaccuracy produced little effect on distance estimation even when true distances were as large as 2.0. When scaled to percent identity (the proportion of aligned sites that contain the same nucleotides in the paired sequences), the curves for the different substitution models become congruent (Figure 3B). The robustness of these estimates appears to be related to the inflation of sequence identity (Figure 1C). As long as the true identity is greater than 50%, there is little inflation in the estimated identity due to alignment (even when the alignment is largely wrong). This translates to relatively little error in the estimation of distance, because distance estimates are based solely on the observed proportions of sites that differ among the sequences; JC distance is based on the overall count, Tamura-Nei distances partition the count into transversions and purine and pyrimidine transitions. Since these counts are being estimated reasonably accurately (even though the specific sites are wrong) the distance estimates are also reasonably accurate. The 50% barrier for distance estimate accuracy was also reported by [10] using a least-squares approach to estimating *P*-distance.

It may be possible to reduce the sequence identity inflation by changing the gap and mismatch penalties (or using more sophisticated methods of alignment); these changes would also alter the accuracy of the alignment. The purpose of this study was not to test the best possible way to construct alignments, but rather to examine the effects of typical alignment errors on evolutionary distance estimation from DNA sequences.

**Figure 4:**
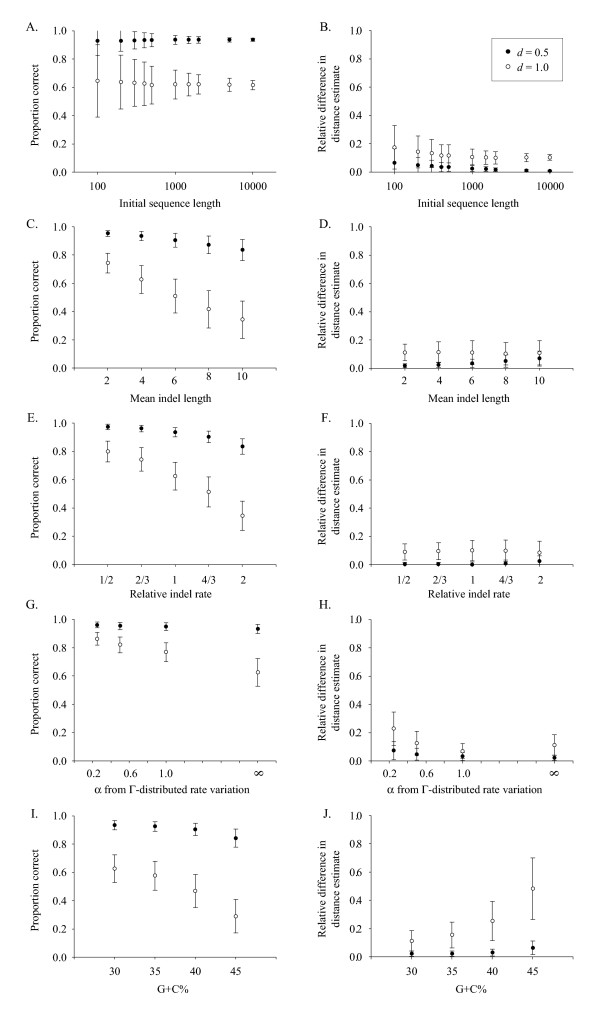
**Effects of parameter changes on alignment accuracy and relative error in evolutionary distance estimation**. Ordinate axes are scaled to match Figure 3. Alignment accuracy (**A, C, E, G, **&**I**) and evolutionary distance estimation (**B, D, F, H, **&**J**). (**A **&**B**) Effect of initial sequence length. (**C **&**D**) Effect of mean insertion and deletion size. (**E **&**F**) Effect of insertion and deletion rate. (**G **&**H**) Effect of intersite rate variation. (**I **&**J**). Effect of nucleotide frequency bias (G+C content). Error bars represent ± one standard deviation. Black and white points represent HKY simulations with expected distances of 0.5 and 1.0, respectively.

The effects of evolutionary parameters on alignment and distance estimation accuracy varied by parameter, but one general observation is that when the value of a specific parameter has an effect, the effect is amplified when the true distance between the pair of sequences is larger (Figure 4). While sequence length generally affects the accuracy of evolutionary distance estimates [9], there was no interaction between sequence length and the effect of alignment accuracy on evolutionary distance estimation. The mean alignment accuracy was unaffected by sequence length, although standard deviations were much reduced for longer sequences (Figure 4A). On the other hand, distance estimates became marginally better with longer sequences (Figure 4B). In contrast to sequence length, increasing both indel size and rate had large effects on alignment accuracy (Figures 4C, E). Evolutionary distance estimation, however, was unaffected by changes in these parameters (Figures 4D, F), showing a lack of association between alignment accuracy and evolutionary distance estimation (i.e., Figure 4 shows a 40% decrease in alignment accuracy with essentially no corresponding change in the accuracy of estimated evolutionary distances).

Changing the α parameter of the Γ-distributed rate variation had an easily predictable effect on alignment accuracy given the results in Figures 2,3. Decreasing α, increases the magnitude of intersite rate variation (an α of infinity indicates equal rates among all sites); thus, decreasing α will increase the proportion of identical sites found among the paired sequences since substitutions will occur at a fewer sites. As already shown, increased identity among the sequences leads to increased accuracy of alignment, a result confirmed in Figure 4G. Accuracy of distance estimates appear driven by the sensitivity of the results to proper specification of the site rate distribution [9], and a relationship with alignment accuracy is uncertain. Estimation of evolutionary distance with intersite rate variation substitution models requires user specification of the Γ-distribution shape parameter α. There are no established methods for estimating α from only a pair of sequences (all described methods require 3 or more sequences). The effect of alignment accuracy on distance estimation is dependent on the accuracy of α (results not shown). When α is underestimated (i.e., intersite rate variation is less than predicted), evolutionary distance will be underestimated for both the true and hypothesized alignments, but the difference between these estimates is reduced (relative to the correct specification of α). When α is overestimated (i.e., intersite rate variation is more than predicted), evolutionary distance will be overestimated for both the true and hypothesized alignments and the difference between these estimates is accentuated.

Not surprisingly, nucleotide frequency biases have a large effect on the accuracy of both alignment and distance estimation (Figures 4I–J). Alignment accuracy decreases with increasing nucleotide frequency bias due to the increased probability of false homology (Figure 4I). A corresponding increase in the error of distance estimation is seen (Figure 4J), but the lack of alignment accuracy cannot necessarily be considered causal. Note the contrast between this result and that for indel rate and size (Figures 4C–F). Increased indel rate and size show a similar magnitude of effect on alignment accuracy as nucleotide frequency, but without the corresponding change in distance estimation. This emphasizes the independence of distance estimation to alignment accuracy for moderate evolutionary divergences.

Overall, these results are somewhat encouraging, particularly when one considers that the more realistic substitution models (i.e., HKY + Γ in this study) are more robust to alignment error for much longer evolutionary distances. However, the robustness of these distance estimates is extremely context dependent. Whether a 10% error in distance estimate is large or small depends on the questions being asked as well as the relative distances of other sequence pairs being analyzed.

Some of the general results in this study have been reported previously [10,11,27], but the present study differs from these in the inclusion of more complicated substitution models (HKY + Γ vs. JC) and distance estimates (Tamura-Nei vs. *P*-distance), as well as a somewhat different approach to sequence alignment. Clustal is among the most commonly used alignment programs and implements a variation of the most commonly used pair wise alignment method, the Needleman-Wunsch algorithm [28]. Algorithms which make statistical estimates of alignment, either maximum likelihood or Bayesian [27,29-32], may also incorporate evolutionary distance estimation, sometimes estimating distances over the alignment probability landscape [12]. These methods may be more accurate than Clustal and thus the relationships between alignment accuracy and distance estimation may be very different for these approaches than those described within this study [10,11,27]. One goal of this study was to profile alignment and distance estimation errors as commonly used by the bioinformatics and genomics community; the methods I employed in the present work are much more commonly used that are the statistical alignment procedures.

The performed simulations represent a global alignment condition (there were no rearrangements which would change homology of the overall sequences) and thus focused on global alignment. Local alignment programs and algorithms, such as BlastZ [33] or Dialign [34], implicitly assume that subsections of the sequences are simply not homologous (or that homologous regions occur in different orders). By finding only conserved regions, local alignment algorithms essentially decrease the probability of false positives (aligned sites which are not truly homologous) while increasing the number of false negatives (unaligned sites which are truly homologous). Thus, within the aligned regions, local alignment may be expected to be more accurate than global alignment, but also may lead to underestimates of evolutionary distances since the poorly conserved homologous regions will likely be excluded from the alignment. The tradeoffs between local and global alignment with respect to distance estimation need to be explored in some depth.

Because alignment error appears to be somewhat underappreciated by the genomics community, the alignment error profiles are in-and-of themselves interesting. While it is generally known that sequences become difficult to align as they diverge (e.g., [14,15]), the precipitous decline in accuracy (Figure 1) has only recently been profiled through simulation [26]. Not surprisingly, the exact nature of these curves appears to be highly dependent on indel size and rate (Figure 4). To some extent, the alignment accuracy profiled in Figure 1 can be viewed as a best-case-scenario since the simulation parameters could be considered realistic, but otherwise low, values. As insertion and deletion events increase in size and rate, alignment accuracy, particularly for more divergent sequences will decline precipitously. It is certainly possible that additional accuracy may be recovered by use of different alignment algorithms or better optimization of the alignment parameters.

## Conclusion

In this study, I've shown evolutionary distance estimation to be somewhat robust to errors in alignment for moderate divergences (>50% identity). Other uses of aligned data, including for example, identification of conserved sites relative to exploration of genetic disease [35,36], are likely to be highly dependent on the accuracy of alignment and even a tiny error may have a large effect on the results. Different alignments are known to lead to different hypotheses in phylogenetic analysis [18,19]; how various phylogenetic methods respond to alignment error is an open question in need of future study.

## Methods

Three large sets of simulations were conducted, each differing by the nucleotide substitution model: Jukes-Cantor (JC) [37], Hasegawa-Kishino-Yano (HKY) [38], and HKY + Γ distributed site rate variation. A summary of all simulation conditions is found in Table 1. For JC, initial sequences consisted of 1000 random nucleotides, with the expected base composition equal for all nucleotides (i.e., 25% each). Initial sequences were replicated into a pair of independent lineages and allowed to evolve under the JC model of evolution to an expected fixed divergence (the realized number of substitutions were drawn from a Poisson distribution), ranging from 0.02 to 2.0. Insertions and deletions were also allowed to occur, with the expected rate of deletion events being one occurrence every 40 substitutions and the expected rate of insertion events being one occurrence every 100 substitutions (as observed in primates and rodents) [39]. Realized number of insertions and deletions were drawn from a Poisson distribution with mean equal to the expected value. The lengths of individual insertion and deletion events were also chosen from a truncated (so as not to include zero) Poisson distribution with a mean of 4 bases (as observed from primate and rodent lineages) [39,40]. Each simulation condition was replicated 1000 times.

The second set of simulations conducted was identical to the first, except using the HKY model of nucleotide substitution. For this model, initial and expected nucleotide frequencies were π_C _= π_G _= 0.3, π_T _= π_A _= 0.2, and the transition-transversion bias was set to that observed at neutral sites in mammals, κ = 3.6 [41]. The third set of simulations conducted was identical to the second, except allowing rate variation among sites within the sequence, modelled by a gamma distribution with a shape parameter of 1.0 [9].

Sequence length is known to play an important role in evolutionary distance estimation; to test for an interaction between sequence length and alignment accuracy, subsets of the HKY simulations were repeated with initial sequences of 100, 200, 300, 400, 500, 1500, 2000, 5000, and 10000 bases. To test the effect of rate and size of insertions and deletions on distance estimation, subsets of the HKY simulations were repeated with mean indel lengths of 2, 6, 8, and 10 bases (the original simulations had a mean of 4 bases) and with insertion and deletion rates of 1 every 200 (insertion) & 80 (deletion) substitutions (half the original rate), 150 & 60 substitutions (2/3 the original rate), 75 & 30 substitutions (4/3 the original rate), and 50 & 20 substitutions (double the original rate). The effects of nucleotide frequencies (G+C% = 60%, 70%, 80%, and 90%) and gamma-distributed rate variation (α = 0.25, 0.5, 1.0, and ∞) were similarly examined.

For every simulated data set, the fate of each of the original sites was tracked and an alignment representing the true homology was constructed for each data set (that is, the simulation program produced gapped sequences in which all aligned sites were truly homologous). The gaps were removed from the sequences and each data set was then aligned using Clustal W version 1.83 [42] with the default parameters, as is common in high-throughput analysis and comparative studies of this sort [26,34,43-45]. This produced a hypothesized alignment, just as one would obtain from analysis of real data. Clustal is one of the most widely used global alignment programs, particularly for high-throughput genomic analysis, and tends to be among the most accurate [26,46]. While it is possible that another program or algorithm or changing the default Clustal parameters might lead to more accurate alignments, the primary purpose of this study is not to highlight the accuracy of this (or any other) alignment program, but rather to examine the effects of alignment error on evolutionary distance estimation. One could purposefully misalign the sequences by hand, but using a common alignment program allows us to create errors consistent with those found in alignment of real data.

Evolutionary distances between sequence pairs were estimated for both the correct and hypothesized alignments using the Jukes-Cantor [37], Tamura-Nei, and Tamura-Nei + Γ formulas [47], as appropriate.

After the initial analyses, the order of the nucleotides in every simulated sequence was completely randomized to create random sequences with identical nucleotide content as the simulated sequences. The random sequences were also aligned using Clustal.

## Authors' Contributions

MR designed, programmed, executed, and analyzed all parts of this study.

## Figures and Tables

**Table 1:** Summary of all simulation conditions

Model	G+C%	Initial # Sites	κ	α	Insertion/Deletion Rate	Mean Indel Size	True distances simulated
JC	0.5	1000	n/a	n/a	100/40	4	0.02, 0.04, 0.06, 0.08, 0.1, 0.2, 0.3, 0.4, 0.5, 0.6, 0.7, 0.8, 0.9, 1.0, 1.2, 1.4, 1.6, 1.8, 2.0
HKY	0.6	1000	3.6	n/a	100/40	4	0.02, 0.04, 0.06, 0.08, 0.1, 0.2, 0.3, 0.4, 0.5, 0.6, 0.7, 0.8, 0.9, 1.0, 1.2, 1.4, 1.6, 1.8, 2.0
HKY + Γ	0.6	1000	3.6	1.0	100/40	4	0.02, 0.04, 0.06, 0.08, 0.1, 0.2, 0.3, 0.4, 0.5, 0.6, 0.7, 0.8, 0.9, 1.0, 1.2, 1.4, 1.6, 1.8, 2.0
HKY	0.6	100	3.6	n/a	100/40	4	0.5, 1.0
HKY	0.6	200	3.6	n/a	100/40	4	0.5, 1.0
HKY	0.6	300	3.6	n/a	100/40	4	0.5, 1.0
HKY	0.6	400	3.6	n/a	100/40	4	0.5, 1.0
HKY	0.6	500	3.6	n/a	100/40	4	0.5, 1.0
HKY	0.6	1500	3.6	n/a	100/40	4	0.5, 1.0
HKY	0.6	2000	3.6	n/a	100/40	4	0.5, 1.0
HKY	0.6	5000	3.6	n/a	100/40	4	0.5, 1.0
HKY	0.6	10000	3.6	n/a	100/40	4	0.5, 1.0
HKY	0.6	1000	3.6	n/a	100/40	2	0.5, 1.0
HKY	0.6	1000	3.6	n/a	100/40	6	0.5, 1.0
HKY	0.6	1000	3.6	n/a	100/40	8	0.5, 1.0
HKY	0.6	1000	3.6	n/a	100/40	10	0.5, 1.0
HKY	0.6	1000	3.6	n/a	200/80	4	0.5, 1.0
HKY	0.6	1000	3.6	n/a	150/60	4	0.5, 1.0
HKY	0.6	1000	3.6	n/a	75/30	4	0.5, 1.0
HKY	0.6	1000	3.6	n/a	50/20	4	0.5, 1.0
HKY	0.7	1000	3.6	n/a	100/40	4	0.5, 1.0
HKY	0.8	1000	3.6	n/a	100/40	4	0.5, 1.0
HKY	0.9	1000	3.6	n/a	100/40	4	0.5, 1.0
HKY + Γ	0.6	1000	3.6	0.25	100/40	4	0.5, 1.0
HKY + Γ	0.6	1000	3.6	0.5	100/40	4	0.5, 1.0

κ is the transition/transversion bias. α is the shape parameter for Γ-distributed intersite rate variation. Insertion/Deletion Rate is relative to the point mutation rate, i.e., a rate of 100/40 indicates 1 insertion every 100 point mutations and 1 deletion every 40 point mutations. Each simulation condition was replicated 1000 times.
